# Possible involvement of p60-S6K1 in accelerating RPS6 phosphorylation for rapid recovery from skeletal muscle disuse atrophy

**DOI:** 10.1186/s42826-025-00250-w

**Published:** 2025-09-10

**Authors:** Takao Inoue, Yuji Kanazawa, Nobuyuki Mizuguchi, Osamu Maenishi, Masatomo Kimura, Man Hagiyama, Azusa Yoneshige, Takaaki Chikugo, Tatsuki Itoh, Takao Satou, Akihiko Ito

**Affiliations:** 1 Department of Pathology, Faculty of Medicine, Kindai University, 377-2 Ohno-Higashi, Osaka-Sayama, Osaka, 589-8511 Japan; 2 Department of Physical Therapy, Faculty of Health and Medical Sciences, Hokuriku University, Ishikawa, Japan; 3 Kindai University Life Science Research Institute, Osaka, Japan; 4 Department of Diagnostic Pathology, Kindai University Hospital, Osaka, Japan; 5 Department of Diagnostic Pathology, Hashimoto Municipal Hospital, Wakayama, Japan; 6 Department of Food Science and Nutrition, Faculty of Agriculture, Kindai University, Nara, Japan

**Keywords:** Disuse atrophy, Delayed muscle recovery, SHRSP, Hypertension, P60-S6K1

## Abstract

**Background:**

Stroke-prone spontaneously hypertensive rats (SHRSP) exhibit slow-twitch muscle-specific hypotrophy compared with normotensive Wistar-Kyoto rats (WKY). Because slow-twitch muscles are prone to disuse atrophy, SHRSP may experience both disuse atrophy and impaired recovery from it. This study investigated the response of SHRSP to disuse atrophy and subsequent recovery, using WKY as a control.

**Results:**

WKY and SHRSP were subjected to a 7-day tail suspension followed by reloading for 1, 3, and 7 days. The soleus of WKY and SHRSP showed similar atrophic rates following tail suspension; however, the recovery after reloading was delayed in SHRSP. Moreover, WKY, but not SHRSP, exhibited sarcomere structure disruption after tail suspension, followed by necrosis, inflammatory cell infiltration, and edema upon reloading. Phosphorylation of ribosomal protein S6 (RPS6), an indicator of protein translation, was significantly higher in tail-suspended WKY—but not SHRSP—than those in non-tail-suspended groups after reloading. p70-S6 kinase 1 (S6K1), an upstream protein of RPS6, was phosphorylated at Thr389 in a mechanistic target of rapamycin complex 1-dependent manner to the same extent in both WKY and SHRSP; however, the expression of p60-S6K1—a shorter isoform of p70-S6K1 that activates RPS6 without p60-S6K1 phosphorylation—significantly increased only in tail-suspended WKY compared with those in non-tail-suspended WKY and tail-suspended SHRSP. Previously, p60-S6K1 protein expression was thought to result from an alternative translation of the full-length S6K1 transcript that also produces other S6K1 isoforms. However, recent studies have identified a p60-S6K1-specific transcript, and our PCR results showed that this p60-S6K1-specific transcript, but not the full-length S6K1 transcript, was significantly increased only in tail-suspended WKY corresponding with the increase of p60-S6K1 protein expression.

**Conclusions:**

SHRSP exhibited different phenotypes in disuse atrophy and recovery from it compared with WKY, which could be related to suppressed RPS6 phosphorylation associated with the lack of upregulation in p60-S6K1 expression. These findings suggest that p60-S6K1, in cooperation with p70-S6K1, activates RPS6 and promotes rapid recovery from disuse atrophy by enhancing the transcription of the p60-S6K1-specific transcript. The study also suggests a potential involvement of hypertension in disuse atrophy and its recovery.

**Supplementary Information:**

The online version contains supplementary material available at 10.1186/s42826-025-00250-w.

## Background

Physical inactivity due to unloading and immobility, such as bed rest and fracture, causes skeletal muscle disuse atrophy [1–5]. One of the most serious problems among older individuals is the difficulty in muscle recovery from disuse atrophy after rehabilitation [6, 7]. Because skeletal muscle is important for maintaining motor function, skeletal muscle atrophy impairs the quality of life due to movement restrictions. In addition, the skeletal muscle also functions as an endocrine organ, secreting myokines, which are bioactive substances [8]. As skeletal muscle constitutes approximately 40% of the body weight, myokines play important roles in maintaining body homeostasis. Disturbances in myokine secretion are associated with a decline in the quality of life and longevity [1, 9, 10]. Therefore, avoiding skeletal muscle atrophy and rapid recovery from atrophy in older individuals are important therapeutic goals.

Sarcopenia, characterized by age-related loss of skeletal muscle mass along with muscle weakness or decreased physical function, is another major concern in older individuals [11]. As reduced daily physical activity is also associated with sarcopenia [12], it is difficult to distinguish between sarcopenia and disuse atrophy caused by physical inactivity. While slow- and fast-twitch muscles are generally affected by disuse atrophy and sarcopenia, respectively [5, 13, 14], the specific atrophied muscle fiber types are not typically distinguished in the diagnosis of sarcopenia.

Hypertension is one of the most common morbidities in older individuals. Cardiovascular disease, primarily caused by hypertension, is a risk factor for sarcopenia [15]. In addition, low muscle mass is a risk factor for hypertension [16, 17]**.** In contrast, lean body mass (LBM) correlates positively with blood pressure [18, 19], while both low and high LBM are positively associated with hypertension [20]. As these controversial results might be attributed to the complicated symptoms in older individuals due to various co-morbidities, it is necessary to eliminate other factors to confirm the relationship between skeletal muscle and hypertension and hypertension-related diseases.

Spontaneously hypertensive rats (SHR) and stroke-prone SHR (SHRSP), which show hypertension-related symptoms starting at a young age, are essential human hypertension models [21–23]. These hypertensive rat models are suitable for investigating the relationship between skeletal muscle and hypertension and hypertension-related diseases, excluding other factors such as age-related sarcopenia. We have previously reported that normotensive Wistar-Kyoto rats (WKY), SHR, and SHRSP show a blood pressure-dependent decrease in body weight [24]. Furthermore, SHRSP exhibit slow-twitch-specific hypotrophy [25], a phenotype different from that of age-related sarcopenia [26]. We hypothesized that hypertensive SHRSP might be affected by disuse atrophy, recovery from disuse atrophy, or both because slow-twitch skeletal muscles are more susceptible to disuse atrophy than fast-twitch ones [14]. Although SHR have recently been employed for muscle atrophy caused by tail suspension, the study mainly focused on atrophic phenotypes, and muscle recovery was not investigated [27]. Thus, the relationship between hypertension or hypertension-related diseases and disuse atrophy, as well as recovery from disuse atrophy, remains poorly understood. Therefore, this study explores whether SHRSP show different phenotypes against disuse atrophy and recovery compared with WKY, aiming to evaluate the potential involvement of hypertension and its related conditions. Moreover, the underlying mechanisms were investigated through a comparative analysis between WKY and SHRSP.

## Methods

### Ethical approval

All experimental protocols conformed to the guidelines published in the “Guide for the Care and Use of Laboratory Animals” of the US National Institutes of Health (8th edition, revised 2011) and were approved by the Institutional Animal Experimentation Committee of Kindai University Faculty of Medicine (approval number: KAME-2022–059).

### Tail suspension

Male WKY and SHRSP maintained at the Kindai University Life Science Research Institution were used at 12 weeks of age. All animals were housed in the animal center under constant temperature (22 °C ± 1 °C) and humidity (50% ± 10%) in a 12:12**-**h light**–**dark cycle and were fed a Funabashi SP diet (Funabashi Farm, Chiba, Japan) and tap water ad libitum. Tail suspension was performed as previously described, with free access to food and water [27, 28]. The rats were divided into tail-suspended and non-tail-suspended groups. Tail suspension was performed for 7 days (designated as R0d), and free movement after release was allowed for 1, 3, and 7 days (designated as R1d, R3d, and R7d, respectively; *n* = 6 for each group). The assignment of rats in the non-tail-suspended group was the same as that in the tail-suspended group. Blood pressure was measured as previously described [25]. Briefly, rats were exposed to 38 °C for 10 min, and the blood pressure was measured using the tail-cuff method without anesthesia. Rats were anesthetized by inhalation of 4% isoflurane for induction and 1.5%–3% isoflurane for maintenance and then euthanized by exsanguination. The soleus, gastrocnemius, plantaris, and tibialis anterior muscle were weighed. Skeletal muscle tissues were prepared and stored for histological and protein expression analyses as described previously [25].

### Antibodies

Anti-dystrophin (sc-73592) and anti-p70 S6 kinase α (sc-8418) antibodies were purchased from Santa Cruz Biotechnology, Inc. (Dallas, TX, USA). Anti-phospho-p70 S6 kinase (Thr389) against ribosomal protein S6 kinase beta-1 (S6K1) (#9205), anti-S6 ribosomal protein (RPS6) (#2217), and anti-phospho-RPS6 (Ser235/236) (#4858) were purchased from Cell Signaling Technology (Danvers, MA, USA). Anti-GAPDH antibody conjugated to horseradish peroxidase (M171-7) was purchased from Medical & Biological Laboratories (Nagoya, Japan). Mouse and rabbit secondary antibodies conjugated to horseradish peroxidase and mouse IgG-Alexa488 were purchased from Cytiva (Buckinghamshire, UK) and Jackson Laboratory (Bar Harbor, ME, USA), respectively.

### Histological analysis

The soleus muscle was sectioned to a thickness of 10 µm at − 20 °C in a cryostat (Leica CM3050 S, Leica Microsystems GmbH, Wetzlar, Germany). After fixation with 4% paraformaldehyde for 10 min, hematoxylin–eosin (H&E) staining and immunostaining of the sections were conducted. The sections immunostained with anti-dystrophin and anti-mouse IgG Alexa Fluor 488 antibodies were photographed with BZ-X710 (Keyence, Osaka, Japan), and the cross-sectional area (CSA) was measured using the BZ-X analysis software for more than 700 muscle fibers per rat.

### Electron microscopy

One-millimeter sections of the soleus muscle were fixed with 2.5% glutaraldehyde and then treated with osmium tetroxide. Longitudinal tissue sections were embedded in Epon and sliced at a thickness of 90 nm. The sections were stained with 4% uranyl acetate and 1% lead citrate and analyzed using transmission electron microscopy (HT7700; Hitachi, Tokyo, Japan).

### Protein expression and phosphorylation analyses using western blotting

The soleus muscle was homogenized in RIPA buffer (Thermo Fisher Scientific, Waltham, MA, USA) supplemented with a proteinase inhibitor cocktail (Sigma, St. Louis, MO, USA) and a phosphatase inhibitor cocktail (Nacalai Tesque Inc., Kyoto, Japan). Homogenized samples were incubated at 4 °C for 1 h with rotation and subsequently centrifuged at 12,000 × *g* for 15 min at 4 °C. The protein concentration in the supernatant was measured using a BCA protein assay kit (Thermo Fisher Scientific), and 200 µg samples (final concentration, 2 µg/µL) were incubated with 4 × sample buffer containing 0.25 M Tris–HCl (pH 6.8), 8% (w/v) SDS, 40% (v/v) glycerol, 8% (v/v) β-mercaptoethanol, and 0.04% (w/v) bromophenol blue at 95 °C for 10 min. The proteins (20 µg) were separated using SDS-PAGE (5–20% gradient gel, ATTO, Tokyo, Japan) and electrotransferred onto a polyvinylidene fluoride membrane (Merck-Millipore, Billerica, MA, USA). Western blotting was performed using appropriate antibodies, and signals were detected using a LAS-4010 instrument (Cytiva). The signal intensity was analyzed using the ImageJ software version 1.53 k (National Institute of Health, Bethesda, MD, USA).

### Reverse Transcription Polymerase Chain Reaction (RT-PCR)

RNA from R1d soleus muscle was isolated using the RNeasy Plus Universal Mini Kit (Qiagen, Germantown, MD, USA). RNA concentrations were measured using a NanoDrop One spectrophotometer (Thermo Fisher Scientific). cDNA was synthesized from 1 µg RNA using the SuperScript IV First-Strand cDNA Synthesis System (Thermo Fisher Scientific) with random hexamer primers. RT-PCR was conducted with KOD FX Neo polymerase (TOYOBO, Osaka, Japan) using specific primers (Supplementary Table 1) under the PCR conditions described in Supplementary Table 2.

### Statistical analyses

Statistical analyses were performed using the R software version 4.1.1 (R Foundation for Statistical Computing, Vienna, Austria, https://www.r-project.org/). First, the data obtained were analyzed using the Shapiro–Wilk normality test to assess the applicability of parametric or nonparametric analyses. For parametric analysis, the *F*-test was performed to decide on using Student’s or Welch’s *t*-test. For nonparametric analysis, the Mann–Whitney *U*-test was used. Multiple comparisons for soleus weight/body weight and western blotting data were conducted with EZR version 1.68 (Jichi Medical School, https://www.jichi.ac.jp/saitama-sct/SaitamaHP.files/ statmed.html). Tukey and Steel–Dwass tests were used for parametric and nonparametric analyses, respectively. A value of *p* < 0.05 was considered statistically significant.

## Results

### Effect of tail suspension and reloading on body weight, blood pressure, and weight of skeletal muscle of lower legs of WKY and SHRSP

**Fig. 1:**
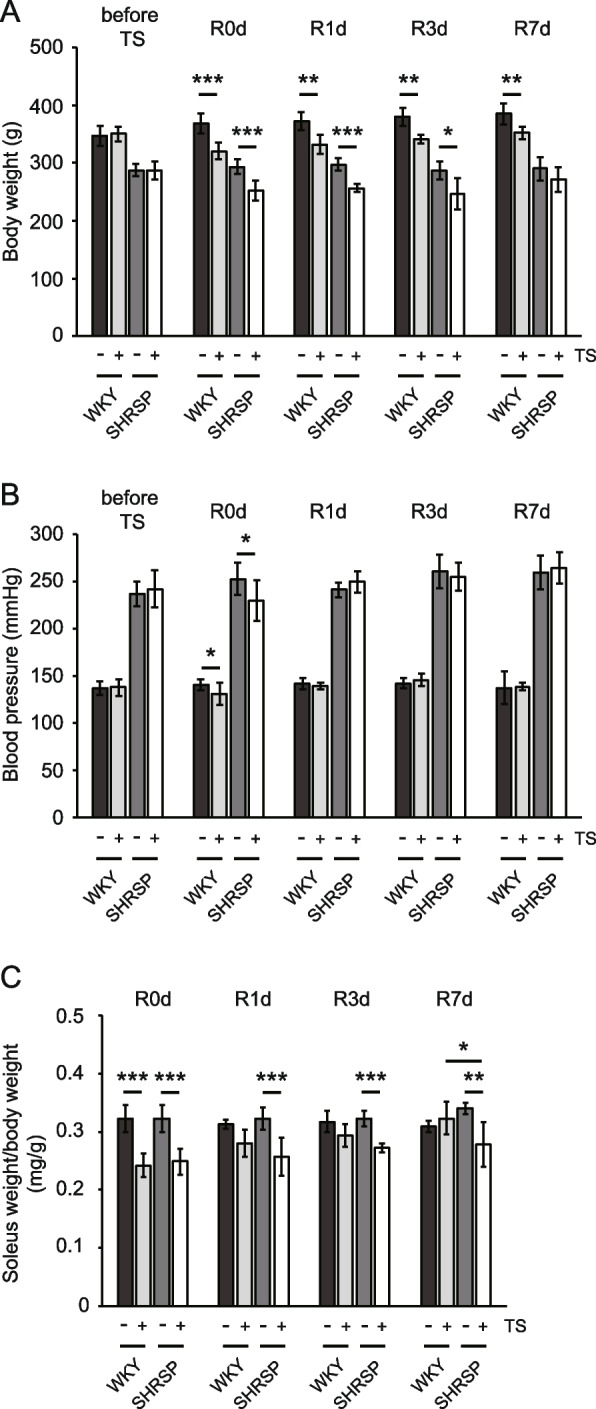
Changes in body weight, blood pressure, and soleus weight/body weight by tail suspension and reloading. **A** Body weight, (**B**) blood pressure, and (**C**) soleus weight/body weight ratio in rats subjected to tail suspension for 7 days followed by reloading. Data are presented as mean ± SD; before TS; just before tail suspension, R0d; tail suspension for 7 days, R1d–R7d; days after reloading, WKY: Wistar-Kyoto rats, SHRSP: stroke-prone spontaneously hypertensive rats, TS: tail suspension. *p < 0.05 **p < 0.01, and ***p < 0.001

Tail suspension for 7 days caused significant body weight loss in both WKY and SHRSP groups compared with their respective non-tail-suspended groups (Table 1). The rate of body weight loss (tail suspension vs. non-tail suspension) between the WKY and SHRSP groups was not significantly different (Table 2). The decrease in body weight induced by tail suspension gradually recovered in both the WKY and SHRSP groups after reloading (Fig. 1A). The body weight between non-tail-suspended and tail-suspended WKY differed significantly at R7d (Fig. 1A). However, the body weight gains from R0d to R7d in the WKY and SHRSP tail-suspended groups were 109.8% ± 3.4% and 107.8% ± 8.4%, respectively, with no significant difference (*p* = 0.595). Blood pressure was significantly decreased in the WKY and SHRSP groups upon tail suspension (Table 1), but the rate of decrease in blood pressure between WKY and SHRSP was not significantly different (Table 2). The decrease in blood pressure in WKY and SHRSP induced by tail suspension recovered on and after the day after reloading (R1d to R7d) (Fig. 1B). The skeletal muscles of the lower leg, namely, the soleus, gastrocnemius, plantaris, and tibialis anterior, showed a significant decrease in weight in the tail-suspended groups (Table 1); however, only the soleus (WKY and SHRSP) and gastrocnemius (WKY) weights normalized against body weight were significantly reduced compared with those in the non-tail-suspended groups (Table 1). The weight of gastrocnemius from SHRSP showed a decreasing trend. These suggest that skeletal muscles with an elevated proportion of slow muscle fibers were considerably affected by disuse atrophy caused by tail suspension for 7 days, as previously reported [14]. Therefore, we focused on the soleus to further investigate the recovery from disuse atrophy. The atrophic rate in soleus weight normalized against body weight (soleus weight/BW) between WKY and SHRSP did not differ significantly (Table 2), indicating that in the soleus, the degree of atrophy was the same irrespective of hypertension or hypertension-related diseases. After reloading, the soleus weight/BW in tail-suspended WKY groups increased rapidly, and no significant differences were observed from R1d to R7d compared with those in the non-tail-suspended groups (Fig. 1C). In contrast, the soleus weight/BW in the SHRSP groups was slightly increased and exhibited a significant difference compared with the non-tail-suspended groups during the experimental period. Furthermore, at R7d, the soleus weight/BW between the WKY and SHRSP tail-suspended groups showed significant differences. The recovery rates at R7d in WKY and SHRSP were 104.8% ± 9.1% and 81.9% ± 11.5%, respectively, with a significant difference (*p* = 0.003), indicating that recovery of soleus weight from disuse atrophy was delayed in SHRSP.

**Table 1:** Effect of tail suspension on body weight, blood pressure, and muscle weight of lower legs

	WKY		*p*-value	SHRSP		*p*-value
	Non-tail suspension	Tail suspension		Non-tail suspension	Tail suspension	
Body weight (g)	369.1 ± 17.7	320.9 ± 14.2	< 0.001	293.7 ± 13.2	252.5 ± 17.3	< 0.001
Blood pressure (mmHg)	139.7 ± 5.7	130.1 ± 12.1	0.025	252.4 ± 17.2	229.4 ± 21.2	7.74E-03
Skeletal muscle (g)						
Soleus	0.115 ± 0.007	0.075 ± 0.006	< 0.001	0.093 ± 0.006	0.063 ± 0.005	< 0.001
Gastrocnemius	1.561 ± 0.036	1.249 ± 0.091	< 0.001	1.305 ± 0.051	1.111 ± 0.072	< 0.001
Plantaris	0.244 ± 0.010	0.202 ± 0.008	< 0.001	0.252 ± 0.011	0.225 ± 0.017	9.36E-03
Tibialis anterior	0.520 ± 0.032	0.473 ± 0.028	0.037	0.516 ± 0.015	0.465 ± 0.025	1.40E-03
SM/BW (mg/g)						
Soleus	0.323 ± 0.023	0.242 ± 0.021	< 0.001	0.323 ± 0.023	0.248 ± 0.022	< 0.001
Gastrocnemius	4.403 ± 0.239	4.017 ± 0.259	0.023	4.523 ± 0.186	4.335 ± 0.134	0.072
Plantaris	0.687 ± 0.044	0.651 ± 0.035	0.150	0.874 ± 0.021	0.879 ± 0.044	0.799
Tibialis anterior	1.465 ± 0.085	1.520 ± 0.068	0.241	1.789 ± 0.074	1.815 ± 0.063	0.524

Data are presented as mean ± SD; *p*-values were calculated using Student’s *t*-test, Welch’s *t*-test, or Man–Whitney *U*-test

*WKY* Wistar-Kyoto rats, *SHRSP *Stroke-prone spontaneously hypertensive rats, *SM* Skeletal muscle, *BW* Body weight

**Table 2:** Effect of tail suspension on body weight loss, blood pressure decrease, and soleus atrophy

	WKY	SHRSP	*p*-value
Body weight loss (%)	13.1 ± 3.9	14.0 ± 5.9	0.497
Blood pressure decrease (%)	6.8 ± 8.6	9.1 ± 8.4	0.515
Soleus weight atrophy (%)	25.0 ± 6.5	23.1 ± 6.8	0.627
Soleus CSA atrophy (%)	29.7 ± 7.0	36.1 ± 5.6	0.115

The body weight loss, blood pressure decrease, and atrophic rates of soleus weight and cross-sectional area (CSA) were calculated from non-tail-suspended and tail-suspended rats at 7-day tail suspension (R0d). Soleus weights were normalized by body weights

Data are presented as mean ± SD; *p*-values were calculated using Student’s *t*-test or Welch’s *t*-test

*WKY* Wistar-Kyoto rats, *SHRSP* Stroke-prone spontaneously hypertensive rats

### Histological and ultrastructural analyses of the soleus

**Fig. 2:**
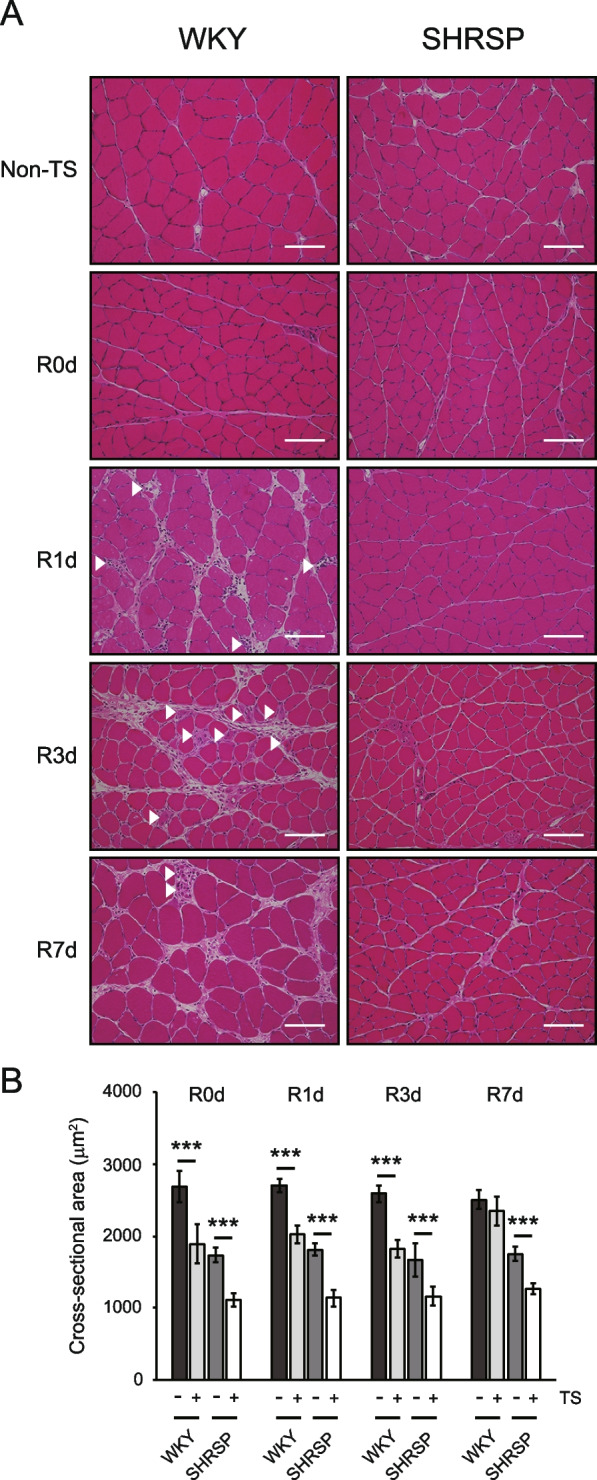
Histological analysis of the soleus muscle subjected to tail suspension and reloading. **A** Hematoxylin–eosin (H&E) staining of muscle sections. Scale bar, 100 μm; arrowheads indicate necrotic fibers or fibers in the regeneration process. **B** Cross-sectional area of soleus muscle fibers. Cross-sectional area was detected using immunostaining with anti-dystrophin antibody and measured for more than 700 fibers per rat (*n* = 6 for each day). Data are presented as mean ± SD. *** *p* < 0.001. WKY: Wistar-Kyoto rats, SHRSP: stroke-prone spontaneously hypertensive rats, TS: tail suspension, Non-TS: non-tail suspension. R0d: tail suspension for 7 days, R1d–R7d: days after reloading

**Fig. 3:**
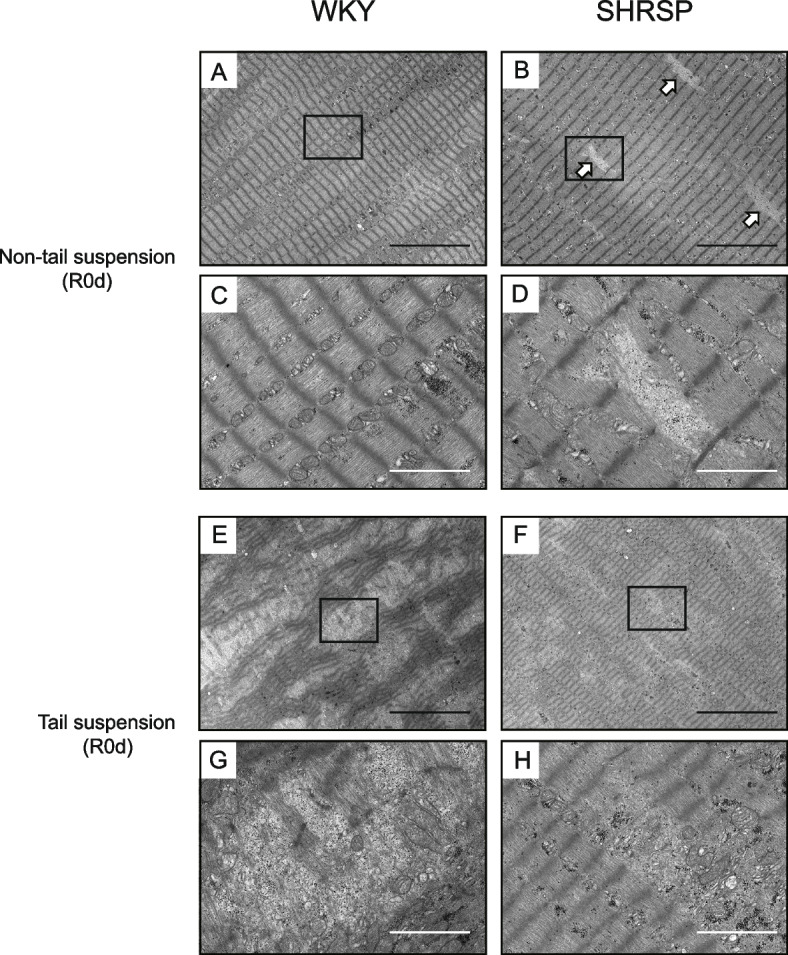
Electron micrographs of the soleus muscle sections subjected to tail suspension. **A**–**D** Sections from non-tail-suspended groups. **C **and **D** show magnified images of the areas marked with squares in (**A**) and (**B**), respectively. Open arrows indicate the sites showing loss of basic structure. **E**–**H** Sections from tail-suspended groups. **G** and **H** show magnified images of the area marked with squares in (**E**) and (**F**), respectively. R0d; tail suspension for 7 days. Scale bar (black) = 10 μm; scale bar (white) = 2 μm. WKY: Wistar-Kyoto rats, SHRSP: stroke-prone spontaneously hypertensive rats

Histological analysis was carried out to examine any differences in the soleus recovery process between WKY and SHRSP. H&E staining of the soleus muscle showed muscle fiber atrophy in both WKY and SHRSP at R0d (Fig. 2A). After reloading, muscle fiber necrosis, fibers in the regeneration process, infiltration of inflammatory cells, and edema were observed in WKY (Fig. 2A, R1d to R7d, arrowheads indicate necrotic fibers or fibers in the regeneration process). In contrast, SHRSP did not exhibit muscle fiber necrosis or inflammation. This indicated that the inflammatory reaction partially caused soleus weight gain in WKY (Fig. 1C). Therefore, we measured the cross-sectional area (CSA) using dystrophin immunostaining to evaluate the recovery of muscle fibers precisely. The soleus from both WKY and SHRSP showed a significantly decreased CSA after tail suspension (Fig. 2B). The difference in the decreasing rate of CSA between WKY and SHRSP was not significant (Table 2). The CSA in WKY was significantly different between the non-tail-suspended and tail-suspended groups until R3d (Fig. 2B), despite no significant difference in the soleus weight/BW between the non-tail-suspended and tail-suspended groups at R1d and R3d (Fig. 1C). This discrepancy could have arisen from weight gain due to the infiltration of inflammatory cells and edema (Fig. 2A). In contrast, the CSA in SHRSP was significantly different during the experimental period, corresponding to the soleus weight/BW (Fig. 1C). The final recovery rate of CSA in WKY and SHRSP at R7d was significantly different, being 93.7% ± 8.3% and 72.4% ± 8.4%, respectively (*p* = 0.001), indicating that WKY were still recovering at R7d, and the soleus weight/BW in WKY at R7d (104.8% ± 9.1%) was due to inflammation. These results suggest that the recovery of muscle fibers, in addition to soleus weight/BW, was delayed in the SHRSP group. To further analyze the differences between WKY and SHRSP, especially for the necrosis of muscle fibers, electron microscopy of soleus samples was performed at R0d. The non-tail-suspended WKY showed a distinct sarcomere structure (Fig. 3A and C). In contrast, the non-tail-suspended group in SHRSP showed a loss of basic structure at some places (Fig. 3B, open arrows, and D). The electron micrograph for WKY in the tail-suspended group showed two types of fibers. One was similar to the tail-suspended SHRSP described below, whereas in the other, the sarcomere structure was extensively disrupted (Fig. 3E and G). SHRSP in the tail-suspended group showed partial and diffuse disruption of the sarcomere structure (Fig. 3F and H). These results indicate that muscle fibers showing a pattern of sarcomere disruption in WKY may be necrotic and induce inflammatory cell infiltration and edema (Fig. 2A).

### Effect of the proteins involved in protein synthesis in the soleus of rats subjected to tail suspension and reloading

**Fig. 4:**
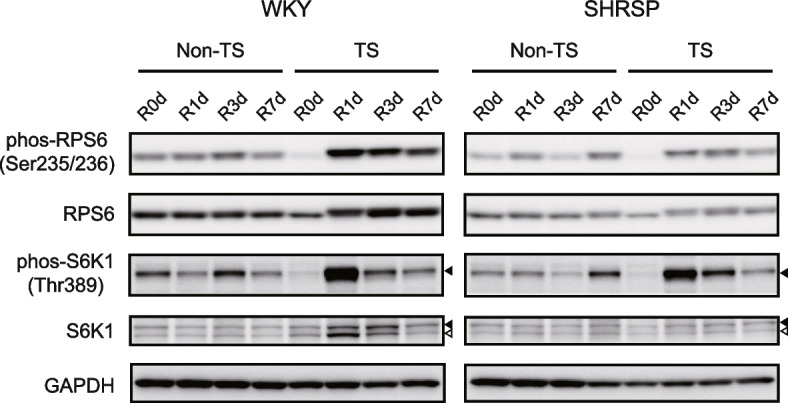
Representative western blotting images of protein synthesis-related proteins subjected to tail suspension and reloading. Closed and open arrowheads indicate p70-S6K1 and p60-S6K1, respectively. WKY: Wistar-Kyoto rats, SHRSP: stroke-prone spontaneously hypertensive rats, Non-TS: non-tail suspension. TS: tail suspension. R0d: tail suspension for 7 days, R1d–R7d: days after reloading, RPS6: ribosomal protein S6, phos-RPS6: phosphorylated RPS6, S6K1: S6 kinase 1, phos-S6K1: phosphorylated S6K1

**Fig. 5:**
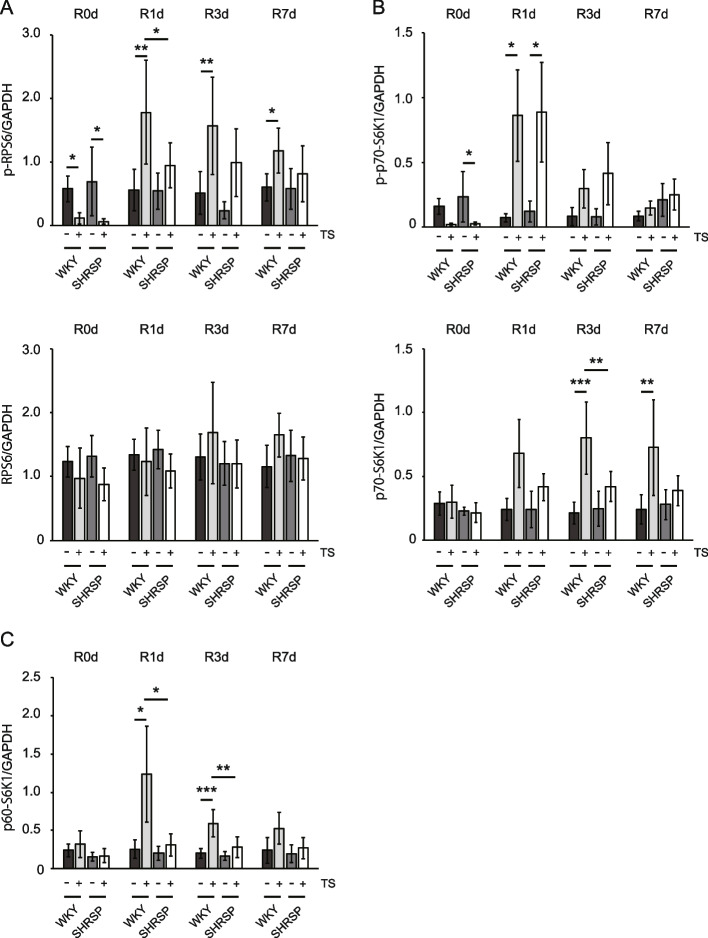
Densitometric analysis for protein synthesis-related proteins subjected to tail suspension and reloading. **A** Densitometric analysis for the band intensities for ribosomal protein S6 (RPS6) phosphorylated at Ser235/236(phos-RPS6) normalized by GAPDH (top panel) and RPS6 normalized by GAPDH (bottom panel). **B** Densitometric analysis for the band intensities for p70-S6 kinase 1 (S6K1) phosphorylated at Thr389 (phos-S6K1) (closed arrowheads in Fig. 4) normalized by GAPDH (top panel) and p70-S6K1(closed arrowheads in Fig. 4) normalized by GAPDH (bottom panel). **C** Densitometric analysis of p60-S6K1 (open arrowheads in Fig. 4) normalized by GAPDH. Data are presented as mean ± SD. * *p* < 0.05, ** *p* < 0.01, and *** *p* < 0.001. WKY: Wistar-Kyoto rats, SHRSP: stroke-prone spontaneously hypertensive rats, TS: tail suspension, R0d: tail suspension for 7 days, R1d–R7d: days after reloading

To evaluate the differences in the recovery from disuse atrophy between WKY and SHRSP, the phosphorylation (and consequent activation) of ribosomal protein S6 (RPS6), which is involved in protein translation, was analyzed to assess protein synthesis in the soleus after tail suspension and reloading. Tail suspension caused a significant decrease in the phosphorylated RPS6 in both the WKY and SHRSP groups (Fig. 4 and Fig. 5A, top panel). After reloading, tail-suspended WKY showed a significant increase in RPS6 phosphorylation compared with non-tail-suspended WKY during the experimental period. In contrast, tail-suspended SHRSP showed an increasing trend, with no significant difference between non-tail-suspended and tail-suspended groups. Additionally, phosphorylated RPS6 in tail-suspended WKY at R1d was significantly higher than that in tail-suspended SHRSP. However, no significant difference was observed in the total RPS6 protein expression level (Fig. 5A, bottom panel). These results indicate that the activation of RPS6 was suppressed in SHRSP during recovery compared with that in WKY. We also examined the phosphorylation of p70-S6 kinase 1 (S6K1), which is upstream of RPS6 and directly phosphorylates RPS6. The phosphorylation of p70-S6K1 at Thr389, which is directly mediated by the mechanistic target of rapamycin complex 1 (mTORC1) [29, 30], was examined. The phosphorylated p70-S6K1 was decreased upon tail suspension, corresponding to the suppression of RPS6 in both the WKY and SHRSP groups (significance only in SHRSP) (Fig. 4, closed arrowheads and Fig. 5B, top panel). After reloading, the phosphorylated p70-S6K1 at R1d showed a significant difference compared with non-tail-suspended groups, but no significant difference was observed between tail-suspended WKY and SHRSP, indicating that the direct activation of p70-S6K1 by mTORC1 during reloading occurred at the same level in both groups. The expression level of p70-S6K1 was significantly increased only in tail-suspended WKY compared with non-tail-suspended WKY at R3d and R7d and with tail-suspended SHRSP at R3d (Fig. 4, closed arrowheads and Fig. 5B, bottom panel). Furthermore, in WKY soleus, but not SHRSP, the expression of p60-S6K1, an alternative shorter isoform of p70-S6K1 that does not require Thr359 phosphorylation (corresponding to Thr389 of p70-S6K1) for RPS6 phosphorylation [31, 32], was significantly increased at R1d and R3d (Fig. 4, open arrowheads and Fig. 5C). This difference was observed not only between non-tail-suspended and tail-suspended groups, but also between WKY and SHRSP tail suspended groups. In addition, p60-S6K1 expression in WKY decreased, accompanied by an increase in p70-S6K1 expression (Fig. 5B, bottom panel and Fig. 5C). These findings indicate that p60-S6K1 and p70-S6K1 may cooperatively participate in rapid recovery from disuse atrophy in WKY, leading to higher activation of RPS6 in WKY than that in SHRSP.

### Gene expression involved in p60-S6K1 protein expression

**Fig. 6:**
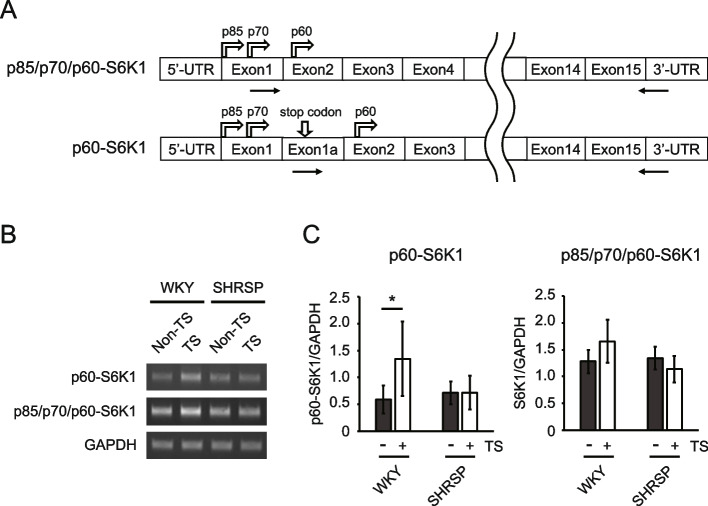
Transcription level of S6 kinase 1 (S6K1) isoforms at 1 day after reloading. **A** Transcript images for p85/p70/p60-S6K1 (upper) and p60-S6K1(lower). Arrows: positions for primers, arrows on exon 1 and 2: positions of the start codon for the isoforms. **B** Representative RT-PCR images. **C** Densitometric analysis of p60-S6K1-specific and p85/p70/p60-S6K1 transcripts normalized by GAPDH. Data are presented as mean ± SD. * *p* < 0.05. UTR: untranslated region, S6K1: S6 kinase 1, WKY: Wistar-Kyoto rats, SHRSP: stroke-prone spontaneously hypertensive rats, Non-TS: non-tail suspension. TS: tail suspension

Western blotting analysis suggested that p60-S6K1 expression is necessary for rapid recovery from disuse atrophy; however, its transcriptional regulation remains poorly understood. Although p60-S6K1 is proposed to be translated by alternative translation from the full-length p85/p70/p60-S6K1 transcript [33], the p60-S6K1-specific transcript exists (Fig. 6A) [34]. The p85/p70/p60-S6K1 transcript has exon 1–15 with 5′- and 3′-untranslated regions (Fig. 6A, the upper transcript). In contrast, the p60-S6K1-specific transcript contains an additional exon, exon 1a, which is part of the intronic region between exons 1 and 2 (Fig. 6A, lower transcript). Exon 1a includes a stop codon that prevents translation of upstream isoforms, thereby enabling translation to initiate at the downstream start codon specific to p60-S6K1 [34]. To understand the mechanism of p60-S6K1 gene expression, RT-PCR was conducted using the soleus from non-tail-suspended and tail-suspended R1d samples from WKY and SHRSP. The expression levels of p60-S6K1-specific and full-length p85/p70/p60-S6K1 transcripts were assessed using specific primers (Fig. 6A, arrows and supplementary Table 1) to obtain almost full-length transcripts and minimize the contamination of other S6K1 isoforms, such as truncated isoforms. In addition, the PCR products corresponding to p60-S6K1 and full-length p85/p70/p60-S6K1 were confirmed by sequencing (data not shown). The expression level of the p60-S6K1-specific transcript, but not that of full-length p85/p70/p60-S6K1 transcript, was significantly increased in tail-suspended WKY (Fig. 6B and C). In contrast, the expression levels of either of these transcripts did not differ significantly between non-tail-suspended and tail-suspended SHRSP groups. These findings suggest that the p60-S6K1-specific transcript plays a crucial role in p60-S6K1 protein expression.

## Discussion

In the present study, we assessed how SHRSP respond to disuse atrophy and recover from disuse atrophy using normotensive WKY as the control. No significant difference in the rate of change in the soleus weight/BW or CSA upon tail suspension was observed for the two groups (Table 2). The rate of change observed in the present study corresponds with that observed in a previous study using Sprague–Dawley rats [35]. However, SHRSP exhibited a significantly delayed recovery from disuse atrophy with regard to the soleus weight/BW and CSA. Histopathological analysis revealed necrosis of the soleus muscle fibers and accompanying infiltration of inflammatory cells during recovery from disuse atrophy in WKY, but not in SHRSP. Fiber necrosis and infiltration of inflammatory cells observed in WKY upon tail suspension followed by reloading appear to be normal physiological responses [35–38]. Tidball et al. showed that inhibiting inflammatory cell (macrophage) function after reloading subsequent to tail suspension caused a delay in skeletal muscle recovery [36]. Macrophages have a protective effect on damaged fibers and facilitate muscle fiber recovery [36, 39]. In addition, intracellular molecules released from damaged or necroptotic muscle fibers activate satellite cells [40, 41]. These findings suggest that muscle fiber necrosis and infiltration of inflammatory cells, especially macrophages, during the recovery process are necessary for rapid recovery. To determine the reason for the absence of fiber necrosis in SHRSP during recovery from disuse atrophy, we performed an electron microscopic analysis of the soleus section from rats subjected to tail suspension. Remarkably, WKY showed complete disruption of sarcomere structure in some muscle fibers, consistent with a previous study [35]. In contrast, the muscle fibers in SHRSP exhibited only partial and diffuse sarcomere disruption. These findings indicate that (1) sarcomere structure disruption observed in WKY is a normal disuse atrophic process, (2) the fibers showing sarcomere disruption may become necrotic after reloading, and (3) WKY and SHRSP have different degradation systems or degrees of disuse atrophy. However, in the present study, we could not elucidate the specific protein degradation system in the two rat models and ascertain whether hypertension or hypertension-related diseases are directly involved in the different protein degradation systems. Further studies are warranted to elucidate the mechanisms underlying differential protein degradation in WKY and SHRSP, as this may contribute to the differences in recovery.

The findings suggest that protein synthesis must be activated for rapid recovery from disuse atrophy. RPS6 phosphorylation was suppressed in SHRSP compared with WKY, corresponding to delayed recovery from disuse atrophy in SHRSP. The upstream protein for RPS6, p70-S6K1, was equally phosphorylated at Thr389 in both groups, indicating equivalent mTORC1-dependent stimulation by reloading. However, WKY showed a significant increase in total p70-S6K1, indicating that the phosphorylation efficiency normalized by total p70-S6K1 in WKY was lower than that in SHRSP (data not shown). This suggests that unphosphorylated p70-S6K1, as well as phosphorylated p70-S6K1, may play an important role in protein synthesis. In addition, the expression of p60-S6K1 increased only in WKY after reloading. p60-S6K1 is a unique S6K1 isoform formed by a 30-amino acid N-terminal truncation of p70-S6K1 [31]. Since the truncated region contains the mTORC1-binding site, p60-S6K1 is not phosphorylated at Thr359 (T389 for p70-S6K1) by mTORC1. This unphosphorylated p60-S6K1 at Thr359 exhibits kinase activity for RPS6 [32]. Our findings demonstrated no phosphorylation of p60-S6K1 at Thr359. Furthermore, p60-S6K1 is marginally expressed in normal cells and not phosphorylated at the mTORC1-dependent site [32], which is consistent with our results. In contrast, p60-S6K1 is highly expressed and phosphorylated at the mTORC1-dependent site in MCF7 cells, a breast cancer cell line, for which the specific underlying mechanism remains unclear. As unphosphorylated p60-S6K1 is involved in cell proliferation and migration [32], increased expression of p60-S6K1, in addition to phosphorylated p70-S6K1, would contribute to the higher activation of RPS6 in WKY. In addition, the increased p60-S6K1 expression induced by reloading appears suitable for rapid protein translation as it avoids the extra energy consumption required for phosphorylation and circumvents several signal transductions. Therefore, further investigation is warranted to ascertain how p60-S6K1 expression is regulated as a possible therapeutic target to support recovery from disuse atrophy.

Moreover, the expression mechanism of p60-S6K1 protein remains unclear. p60-S6K1 was thought to be translated from a full-length p85/p70/p60-transcript by alternative translation [33]. However, evidence from a recent study [34] suggests the presence of a p60-S6K1-specific transcript. Zaiets et al. identified a p60-S6K1 splicing variant in which the extra sequence (exon 1a) is inserted between exon 1 and 2, introducing a stop codon upstream of the p60-S6K1 start codon [34], which facilitates the specific translation of p60-S6K1. RT-PCR results in the present study suggest that the p60-S6K1-specific transcript strongly contributes to the expression of p60-S6K1 protein. Nevertheless, further studies are needed to elucidate the regulatory mechanisms of this transcript to promote rapid recovery from disuse atrophy.

To date, no comprehensive studies have assessed whether hypertension or hypertension-related diseases are associated with disuse atrophy or recovery from it. Nemoto and Goyagi explored tail suspension-induced muscle atrophy in SHR as a model for sarcopenia [27]. The study showed significant decrease in the weights of the extensor digitorum longus (EDL) and soleus in tail-suspended rats, whereas no significant difference was observed for EDL or soleus normalized against body weights compared with non-tail-suspended rats [27]. This lack of a significant difference could be because they used non-tail-suspended Sprague–Dawley rats, not non-tail-suspended SHR, as controls. Furthermore, the CSA of SHR showed a significant difference compared with that of non-tailed Sprague–Dawley rats, possibly due to higher atrophic rates of CSA than that of muscle weight. However, they did not further investigate recovery from muscle atrophy, leaving the recovery process in SHR unclear. Disuse atrophy may closely relate to sarcopenia in older individuals, and distinguishing these two conditions is challenging. Using young hypertensive rats (SHRSP), our study excluded the possibility of sarcopenia from disuse atrophy. Although direct evidence for the relationship between disuse atrophy and recovery from disuse atrophy and hypertension and hypertension-related diseases remains elusive, our findings suggest a potential involvement of hypertension and hypertension-related diseases in disuse atrophy and recovery from it.

## Conclusions

Hypertensive SHRSP showed delayed muscle recovery after disuse atrophy compared with normotensive WKY. This delay is most likely due to the lack of an increase in p60-S6K1, followed by the suppression of RPS6 phosphorylation and insufficient activation of protein synthesis in SHRSP during the recovery. However, it remains unclear whether the difference in protein synthesis between the two rat strains is related to differences in the degradation process, such as sarcomere structure disruption, fiber necrosis, and inflammatory cell infiltration, observed in WKY. Furthermore, we demonstrated that p60-S6K1 is involved in rapid recovery from disuse atrophy via RPS6 activation, and the upregulation of p60-S6K1 could serve as a therapeutic target for rapid recovery from disuse atrophy and possible improvement in rehabilitation and resistance to muscle weight loss due to aging. Thus, elucidating the mechanisms underlying the regulation of p60-S6K1 protein expression, especially the transcription of p60-S6K1-specific transcript, is necessary for the development of therapies against muscle growth disorders. This study offers insights into the mechanisms underlying impaired skeletal muscle recovery in older individuals with co-morbidities, including hypertension and hypertension-related diseases, and suggests that blood pressure control is a potential therapeutic target to support recovery from disuse atrophy, a topic we are currently investigating.

## Supplementary Information

Supplementary Material 1.

Supplementary Material 2.

Supplementary Material 3.

Supplementary Material 4.

Supplementary Material 5.

Supplementary Material 6.

Supplementary Material 7.

## Data Availability

Data will be made available on request.

## Abbreviations

BW: Body weight
RPS6: Ribosomal protein S6
S6K1: S6 kinase 1
SHR: Spontaneously hypertensive rats
SHRSP: Stroke-prone SHR
SM: Skeletal muscle
TS: Tail suspension
WKY: Wistar-Kyoto rats
