# Supplementary material for: Possible involvement of p60-S6K1 in accelerating RPS6 phosphorylation for rapid recovery from skeletal muscle disuse atrophy
Source: Lab Anim Res. 2025 Sep 10;41:20. doi: 10.1186/s42826-025-00250-w (PMC12421747; doi:10.1186/s42826-025-00250-w)
Supplement: Supplementary file 1 — Supplementary Material 1. [file 42826_2025_250_MOESM1_ESM.pdf]

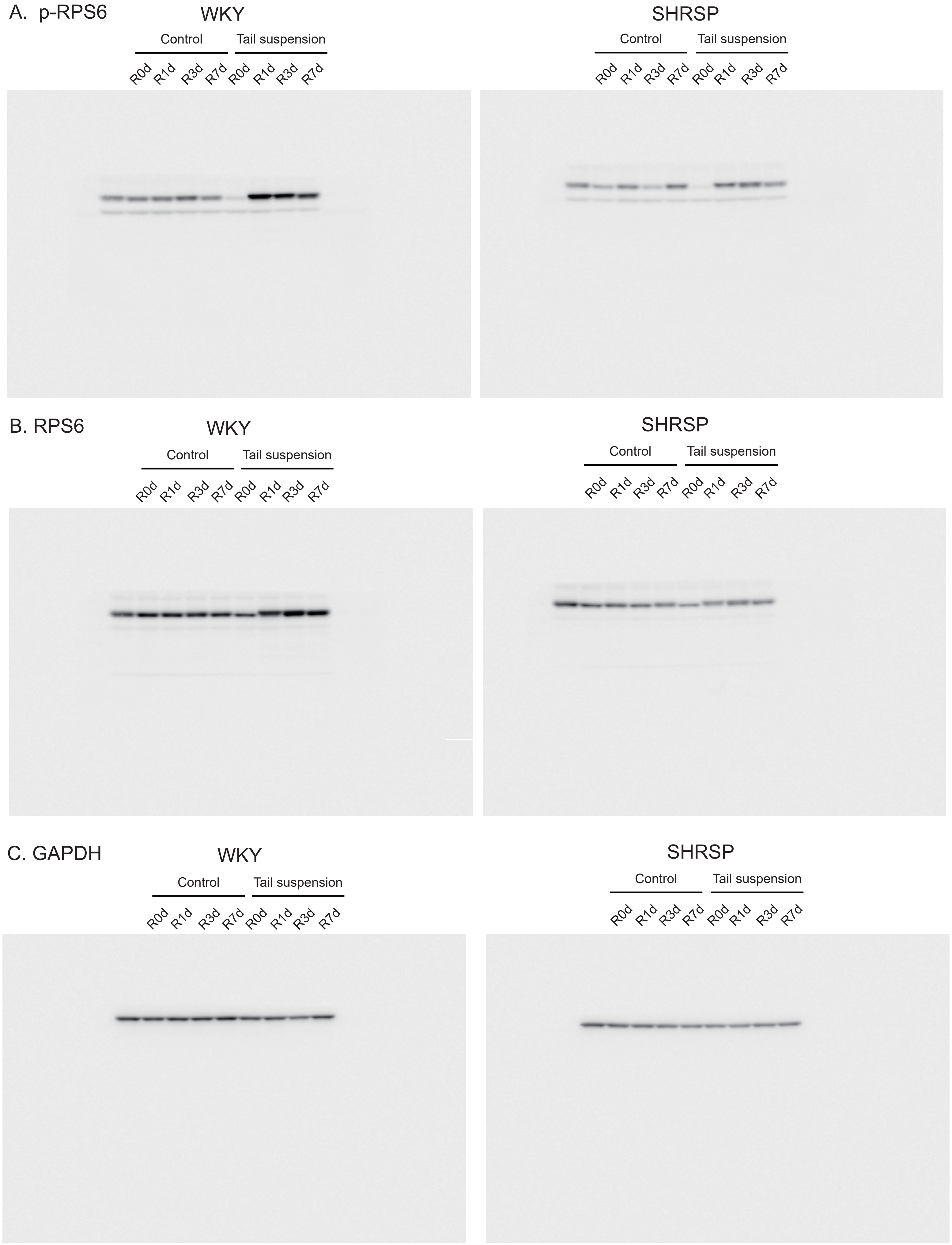

Fig. S1. Original western blot images for phospho-RPS6 (A) and RPS6 (B) shown in Fig. 4, and GAPDH (C) omitted from Fig.4. GAPDH blots used for Fig. S3 were reused for Fig. 4.
