# Supplementary material for: Possible involvement of p60-S6K1 in accelerating RPS6 phosphorylation for rapid recovery from skeletal muscle disuse atrophy
Source: Lab Anim Res. 2025 Sep 10;41:20. doi: 10.1186/s42826-025-00250-w (PMC12421747; doi:10.1186/s42826-025-00250-w)
Supplement: Supplementary file 4 — Supplementary Material 4. [file 42826_2025_250_MOESM4_ESM.pdf]

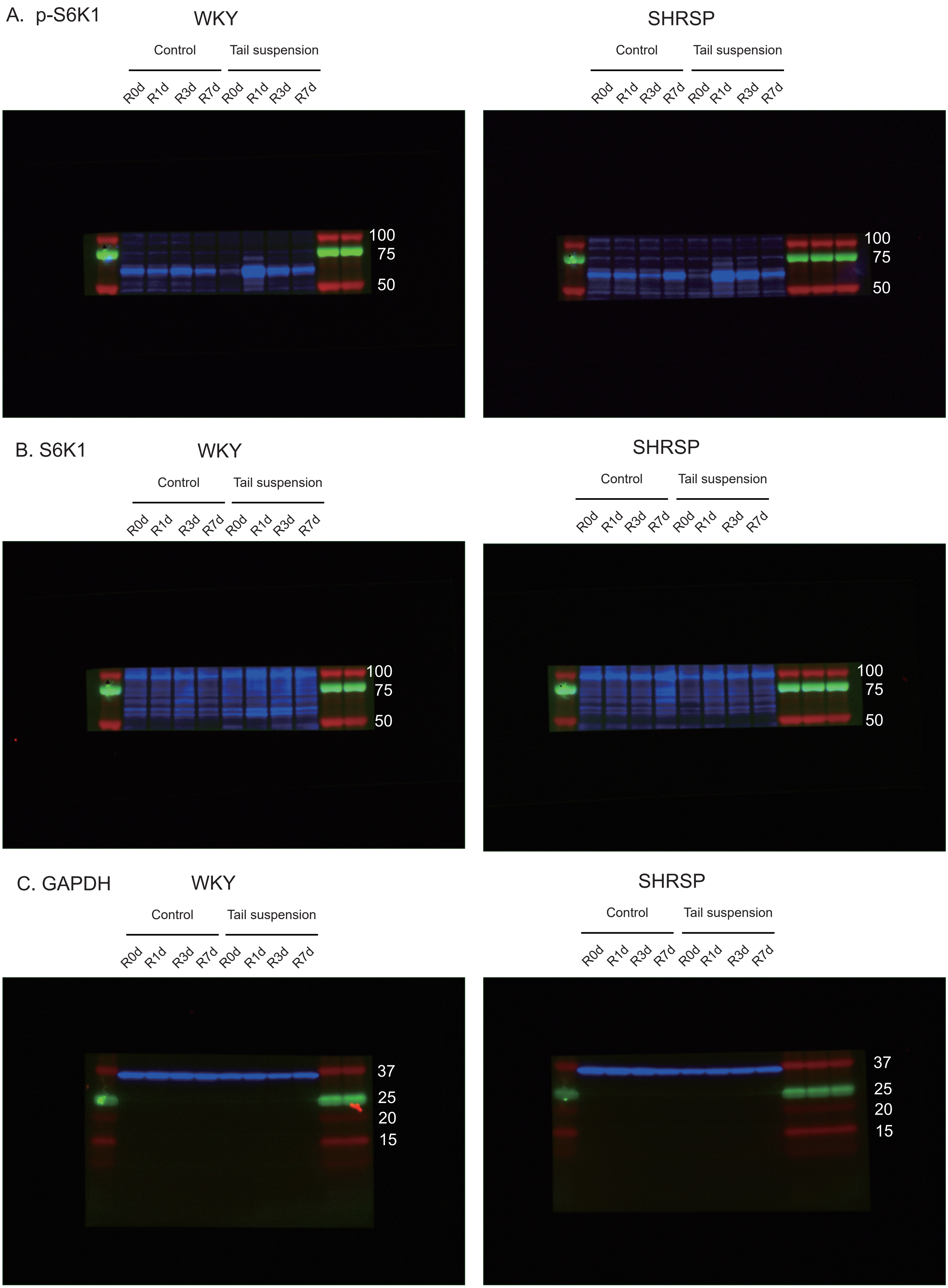

Fig. S4. Merged Original western blot images for phospho-S6K1 (A), S6K1 (B), and GAPDH (C) shown in Fig. S3 with molecular markers.
