# Supplementary material for: Possible involvement of p60-S6K1 in accelerating RPS6 phosphorylation for rapid recovery from skeletal muscle disuse atrophy
Source: Lab Anim Res. 2025 Sep 10;41:20. doi: 10.1186/s42826-025-00250-w (PMC12421747; doi:10.1186/s42826-025-00250-w)
Supplement: Supplementary file 5 — Supplementary Material 5. [file 42826_2025_250_MOESM5_ESM.pdf]

A. p60-S6K1

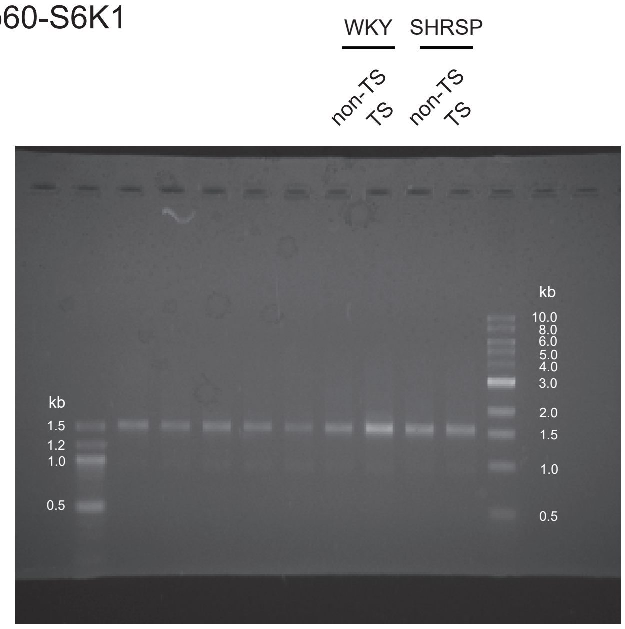

B. p85/p70/p60-S6K1

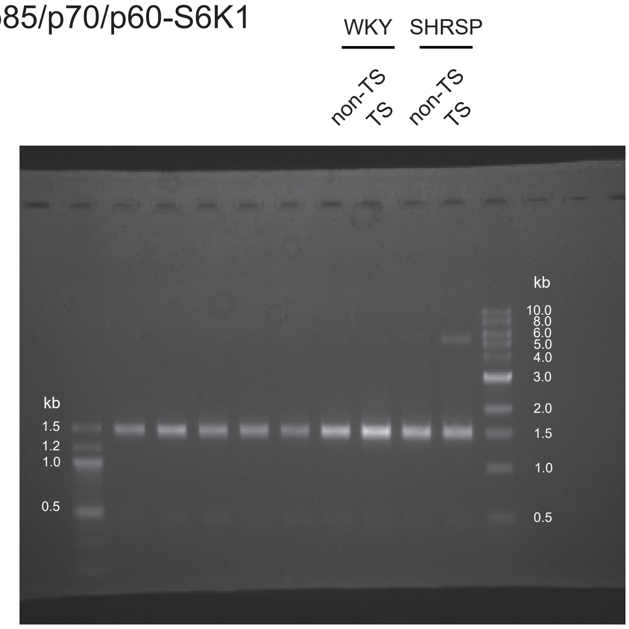

C. GAPDH

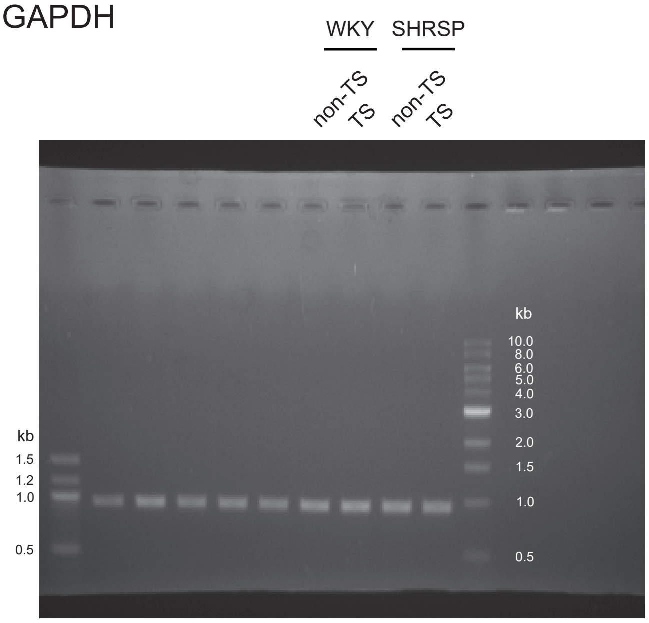

Fig. S3. Original gel images of RT-PCR products for p60-S6K1 (A), p85/p70/p60-S6K1 (B), and GAPDH (C) shown in Fig. 6.
