# Supplementary material for: Possible involvement of p60-S6K1 in accelerating RPS6 phosphorylation for rapid recovery from skeletal muscle disuse atrophy
Source: Lab Anim Res. 2025 Sep 10;41:20. doi: 10.1186/s42826-025-00250-w (PMC12421747; doi:10.1186/s42826-025-00250-w)
Supplement: Supplementary file 6 — Supplementary Material 6. [file 42826_2025_250_MOESM6_ESM.docx]

Supplementary Table 1. Primer set for RT-PCR

| Gene | Forward primer (5′→3′) | Reverse primer (5′→3′) |
| --- | --- | --- |
| P60-S6K1 | GAGTAGCACTGCTGTGACAG | GCATTAATAAAAGCATTGTTTCATCA |
| S6K1 | CAGGCTCTGAGGATGAGCTG | GCATTAATAAAAGCATTGTTTCATCA |
| GAPDH | GCGAGATCCCGCTAACATCA | ATTCGAGAGAAGGGAGGGCT |
|  |  |  |
