# Supplementary material for: Possible involvement of p60-S6K1 in accelerating RPS6 phosphorylation for rapid recovery from skeletal muscle disuse atrophy
Source: Lab Anim Res. 2025 Sep 10;41:20. doi: 10.1186/s42826-025-00250-w (PMC12421747; doi:10.1186/s42826-025-00250-w)
Supplement: Supplementary file 7 — Supplementary Material 7. [file 42826_2025_250_MOESM7_ESM.docx]

Supplementary Table 2. PCR conditions

|  | p60-S6K1 | p85/p70/p60-S6K1 | GAPDH |
| --- | --- | --- | --- |
| 1 | 94 °C, 2 min | 94 °C, 2 min | 94 °C, 2 min |
| 2 | 98 °C, 10 s | 98 °C, 10 s | 98 °C, 10 s |
| 3 | 57 °C, 30 s | 57 °C, 30 s | 57 °C, 30 s |
| 4 | 68 °C, 45 s | 68 °C, 45 s | 68 °C, 45 s |
| 5 | 68 °C, 5 min | 68 °C, 5 min | 68 °C, 5 min |
| Cycles (2 to 4) | 28 | 25 | 20 |
